# Supplementary material for: Exposure-Response and Clinical Outcome Modeling of Inhaled Budesonide/Formoterol Combination in Asthma Patients
Source: Pharmaceutics. 2020 Apr 9;12(4):336. doi: 10.3390/pharmaceutics12040336 (PMC7238265; doi:10.3390/pharmaceutics12040336)
Supplement: Supplementary file 1 [file pharmaceutics-12-00336-s001.pdf]

pharmaceutics

Supplementary Materials: Exposure-Response and Clinical Outcome Modeling of Inhaled Budesonide/Formoterol Combination in Asthma Patients

Hyun-moon Back, Jong Bong Lee, Anhye Kim, Seon-Jong Park, Junyeong Kim, Jung-woo Chae, Seung Soo Sheen, Leonid Kagan, Hae-Sim Park, Young-Min Ye, Hwi-yeol Yun

Secondary endpoints of the clinical study and inclusion/exclusion criteria for study subjects.

Secondary Endpoints

- Comparing the improvement of Asthma Control Test score depending on ADRB2 genotype after inhaling budesonide/formeterol for 12 weeks.
- Comparing the observed pharmacokinetic properties of budesonide and formoterol depending on ADRB2 genotype after repeated inhalation of budesonide/formoterol.
- Comparing changed sputum eosinophils cationic proteins depending on the ADRB2 genotype.
- Comparing the frequency of adverse effects, especially blood potassium and glucose levels and QT interval, depending on ADRB2 genotype.

Inclusion Criteria

The subject who had ADRB2 genotype which are Arg/Arg, Arg/Gly, Gly/Gly.

Moderate asthma patients with age from 20 to 65.

The subject who showed improving more than 12% of FEV1 and 200 mL of volume after inhaling short-acting β2-adrenergic agonists in bronchodilator reversibility test.

The subject who was diagnosed as an asthmatic more than 6 months ago, and used a constant dose of inhaled steroid (alvesco ≤ 320 μg/day, Pulmicort ≤ 1200 μg/day) or inhaled steroid and long-acting β2-adrenergic agonists (seretide ≤ 500 μg/day, symbicort ≤ 320 μg/day) in the last 4 weeks.

The subject who had predicted FEV1 value from 55% to 90%.

Those who were determined to be suitable as subjects by clinical laboratory tests, such as hematology, blood chemistry, and urine tests, which are established and conducted by the doctor in accordance with the characteristics of the drug.

For female subject who was not pregnant at screening.

Those who have received full explanation of this study and agreed to participate in the study in accordance with their free will.

Exclusion Criteria

The subject with a history of respiratory infections within the last 2 weeks.

The subject who had been hospitalized and emergency room for asthma exacerbation within the last 4 weeks, or have received systemic (oral or intravenous) steroids in addition to short-acting β2-adrenergic agonists.

The subject with severe cardiovascular disease (myocardial infarction and unstable angina within the last 6 months).

Those who had taken drugs that may interfere with the study within one week of the study.

The subject with a history of hypersensitivity to the drug.

The subject with excessive caffeine and alcohol intake and smokers (Caffeine > 5 cups/day, alcohol 30g/day, tobacco > 10 cigarettes/day).

The subject who participated in other clinical trials within 2 months.

The subject who donated blood within 1 month.

The subject who had taken drugs which induces or inhibits drug metabolism, such as barbital drugs, within 1 month.

The subject who was unable to refrain from excessive consumption of xanthine beverages (coffee, coke, tea, etc) during the study period.

In the opinion of the investigator, the subject who was not eligible for the study participation for any reason.
